# Stress-responsive nucleus accumbens dopamine D2 receptor-expressing neurons modulate cocaine-induced behavioral sensitization

**DOI:** 10.3389/fncel.2026.1919290

**Published:** 2026-08-12

**Authors:** Byeong Jun Kang, Bokyeong Kim, Minji Kim, Yoonsoo Choi, Eum-Ji Kim, Ja Wook Koo, Ja-Hyun Baik

**Affiliations:** 1 Molecular Neurobiology Laboratory, Department of Life Sciences, School of Life Sciences and Biotechnology, Korea University, Seoul, Republic of Korea; 2 Emotion, Cognition and Behavior Research Group, Korea Brain Research Institute, Daegu, Republic of Korea; 3 Department of Brain Sciences, Daegu Gyeongbuk Institute of Science and Technology, Daegu, Republic of Korea

**Keywords:** activity-dependent labeling system, cocaine, dopamine D2 receptor, nucleus accumbens, optogenetics, stress

## Abstract

Although both stress and cocaine induce robust plasticity within the nucleus accumbens core (NAcc), how these distinct experiences interact at the cellular level to shape behavior remains unclear. Here, we investigated the role of NAcc dopamine D2 receptor-expressing medium spiny neurons (D2R-MSNs) in integrating stress- and drug-induced signals. D2R-MSNs showed distinct activity responses, with increased activity during restraint stress and reduced activity following repeated cocaine exposure. Using the Cre-dependent Robust Activity Marking (cRAM) system, we found that cocaine exposure suppressed the activity of the subpopulation of D2R-MSNs previously recruited by stress. Optogenetic reactivation of these stress-tagged D2R-MSNs during withdrawal attenuated the expression of cocaine-induced behavioral sensitization. Transcriptomic profiling of stress-responsive D2R-MSNs identified cholecystokinin (*Cck*) as a candidate associated with this neuronal manipulation. Subsequent experiments showed that cocaine exposure increased CCK expression in NAcc D2R-MSNs, whereas conditional Cck knockdown reduced the expression of behavioral sensitization. Together, these findings suggest that stress-responsive D2R-MSNs contribute to the regulation of cocaine-induced behavioral sensitization and that CCK signaling within NAcc D2R-MSNs may be involved in this process.

## Introduction

The nucleus accumbens core (NAcc) integrates dopaminergic inputs from the ventral tegmental area with cortical and limbic glutamatergic projections to modulate reward-related behaviors and responses to aversive stimuli (Nestler, 2005; Baik, 2013, 2020). Chronic exposure to psychostimulants, such as cocaine, disrupts these circuits and induces maladaptive plasticity that manifests as behavioral sensitization (Koob and Le Moal, 2001). Various forms of stress potently modulate the acquisition and expression of psychostimulant sensitization, thereby increasing individual vulnerability to addiction (Miczek et al., 2008; Anderson et al., 2019). While recent findings suggest that stress-sensitive neuronal ensembles support these behavioral shifts (Balouek et al., 2023), how stress and cocaine experiences interact within specific NAcc circuits to influence subsequent behaviors remains poorly understood.

The output of the NAcc is primarily governed by dopamine D1 receptor (D1R)- and D2 receptor (D2R)-expressing medium spiny neurons (MSNs), which exert complementary yet opposing actions on behavior (Lobo and Nestler, 2011). While D1R-MSNs typically promote psychostimulant effects, D2R-MSNs provide inhibitory control over reward-motivated actions (Bock et al., 2013). Enhanced glutamatergic drive onto D2R-MSNs serves as a protective mechanism that restrains drug intake and confers individual resilience, whereas their experimental inhibition enhances cocaine motivation (Bock et al., 2013).

Transcriptomic and behavioral evidence suggests that cocaine engages stress-responsive gene programs within D2R-MSNs, and that D2R signaling itself mediates stress-induced alterations in drug sensitization (Sim et al., 2013; Mews et al., 2025). At the cellular level, stress and cocaine can divergently regulate D2R-MSN activity (Vialou et al., 2010; Calipari et al., 2016). However, because previous studies have primarily targeted the broader, unselected D2R-MSN population, it remains unknown how the specific subpopulation of D2R-MSNs directly activated by stress adapts to subsequent cocaine exposure, and whether this distinct functional subpopulation drives behavioral sensitization.

To address this question, we utilized an activity-dependent labeling system combined with optogenetics and transcriptomic profiling to selectively investigate the functional and molecular adaptations of stress-activated D2R-MSNs within the NAcc. Specifically, we examined how subsequent repeated cocaine exposure modulates the activity of this stress-responsive subpopulation and evaluated whether manipulating this subpopulation during withdrawal counteracts the expression of behavioral sensitization. Furthermore, we sought to identify candidate molecular targets uniquely regulated within these neurons to elucidate the molecular mechanisms associated with stress-induced drug neuroadaptations.

## Materials and methods

### Mice

Male Drd2-Cre [B6.FVB(Cg)-Tg(Drd2-cre)ER44Gsat/Mmu cd], Drd2-EGFP [Tg(Drd2-EGFP)S118Gsat/Mmnc] (MMRRC, Davis, CA, USA), and Drd1-tdTomato [B6.Cg-Tg(Drd1a-tdTomato)6Calak/J] (Jackson Laboratory, Bar Harbor, ME, USA) BAC transgenic mice were used. Behavioral experiments were performed using mice at 10–12 weeks of age. Mice were individually housed under standard conditions (12-h light/dark cycle; lights on at 07:00) with ad libitum access to food and water. All animal procedures were approved by the Institutional Animal Care and Use Committee (IACUC) of Korea University (Approval No. KUIACUC-2025-0017). Mice were randomly allocated to experimental and control groups prior to pharmacological, optogenetic, or viral manipulations.

### Viral vector preparation

The following viral vectors were utilized: AAV-EF1a-DIO-GCaMP6s-P2A-nls-dTomato (Addgene #51082) and pAAV-RAM-d2TTA::TRE-FLEX-dTomato-WPREpA (hereafter referred to as AAV-cRAM-dTomato; Addgene #84468). The pAAV-RAM-d2TTA::TRE-FLEX-ChR2-eYFP-WPREpA (AAV-cRAM- ChR2-eYFP) vector was also used. To construct AAV-cRAM-ChR2-eYFP, restriction enzyme cloning was performed. The dTomato sequence in the pAAV-RAM-d2TTA::TRE-FLEX-dTomato-WPREpA backbone was replaced with a ChR2-eYFP fragment. This fragment was isolated from pAAV-EF1a-DIO-hChR2(H134R)-EYFP-WPRE (provided by Karl Deisseroth; Addgene: #20298). Adeno-associated viruses (AAVs) were generated in human embryonic kidney 293T (HEK293T) cells. Cells were transfected with pAAV-DJ and pHelper plasmids using jetPEI (QBiogene, Montreal, QC, Canada). At 48 h post-transfection, the cells were harvested. Cell lysis was performed using 0.5% sodium deoxycholate and benzonase (Sigma-Aldrich, St. Louis, MO, USA). Viral particles were subsequently purified using HiTrap heparin affinity columns (GE Healthcare, Chicago, IL, USA). The purified viruses were concentrated using Amicon Ultra-15 filters (100 kDa cutoff, Millipore, Billerica, MA, USA). The final viral titer ranged from 3 to 6 × 10^12^ particles/mL. Aliquots were stored at −80 °C.

### Generation of AAV-DIO-DSE-mCherry-PSE-shCCK

The AAV-DIO-DSE-mCherry-PSE-shCCK vector was constructed to achieve conditional Cck knockdown. The pDIO-DSE-mCherry-PSE-MCS plasmid (Addgene #129669) was utilized as the backbone. The shRNA targeted the specific Cck sequence 5′-TCAGTGACTCCCAGACCTAATGTT-3′. A miR-133 loop was incorporated into the design to minimize off-target effects. The oligonucleotides used were as follows: Sense, 5′-CTA GGT CAG TGA CTC CCA GAC CTA ATG TTC CTG ACC CAA ACA TTA GGT CTG GGA GTC ACT GAT TTT TTG-3′; Antisense, 5′-AAT TCA AAA AAT CAG TGA CTC CCA GAC CTA ATG TTT GGG TCA GGA ACA TTA GGT CTG GGA GTC ACT GAC-3′. AAV-DIO-DSE-mCherry-PSE-shLacZ served as the negative control. For delivery, viruses were bilaterally injected into the NAcc. The stereotaxic coordinates were anteroposterior (AP) +1.7 mm, mediolateral (ML) ± 1.3 mm, and dorsoventral (DV) −4.5 mm. Drd2-Cre mice were used to selectively target D2R-MSNs.

### Restraint stress

Restraint stress was performed using well-ventilated acrylic restrainers. These devices restricted physical movement while allowing normal breathing. For acute restraint stress (ARS), mice were subjected to a single 2-h session. For chronic restraint stress (CRS), mice underwent the 2-h procedure daily for 14 consecutive days.

### Surgical procedures and optic or fiber photometry cannula implantation

Mice were anesthetized with ketamine (10 μL/g) and xylazine (0.05 μL/g) and placed in a stereotaxic frame (David Kopf Instruments, Tujunga, CA, USA). Viral vectors (2 μL per side) were injected bilaterally into the NAcc (AP +1.7, ML ± 1.3, DV -4.5 from bregma) at 0.1 μL/min using a 31G needle. The needle remained in place for 10 min post-injection. Fiber-optic cannulas (200 μm core, NA 0.22; Doric Lenses, Quebec City, QC, Canada) were implanted at AP +1.7, ML ± 1.35, DV -4.5 and secured with skull screws, C&B Superbond (Sun Medical, Shiga, Japan), and dental cement (Dentsply Sirona, York, PA, USA). Mice received carprofen (2.5 mg/kg) and enrofloxacin (2.5 mg/kg) for 3 days post-surgery and were allowed 12–14 days for recovery and viral expression.

### Fiber photometry

AAV-EF1a-DIO-GCaMP6s-P2A-nls-dTomato was unilaterally injected into the NAcc of Drd2-Cre mice (10–12 weeks), followed by unilateral implantation of photometry cannulas (Doric Lenses, Canada). After 3 weeks, calcium transients were recorded using a TDT RZ5P processor in a soundproof booth. Excitation was provided by 465 nm and 405 nm (isosbestic) LEDs, and signals were detected by a photoreceiver (Newport, Irvine, CA, USA; 2151). Recordings included saline/control (Day 1) and cocaine/stress (Day 2) sessions. Data were analyzed using pMAT software (Bruno et al., 2021). The 405 nm signal was fitted to the 465 nm signal to correct for artifacts. Normalized Z-scores were calculated as (Zscore – Zscore*^baseline^*), where Zscore*^baseline^* represents the mean signal during the 30-s pre-treatment period. Quantitative analysis of mean normalized Z-score and the area under the curve (AUC) was performed on the +30s to +180s window. Average Z-scores and AUC were quantified using custom MATLAB (MathWorks, Natick, MA, USA) codes.

### Cocaine sensitization protocol

**FIGURE 1:**
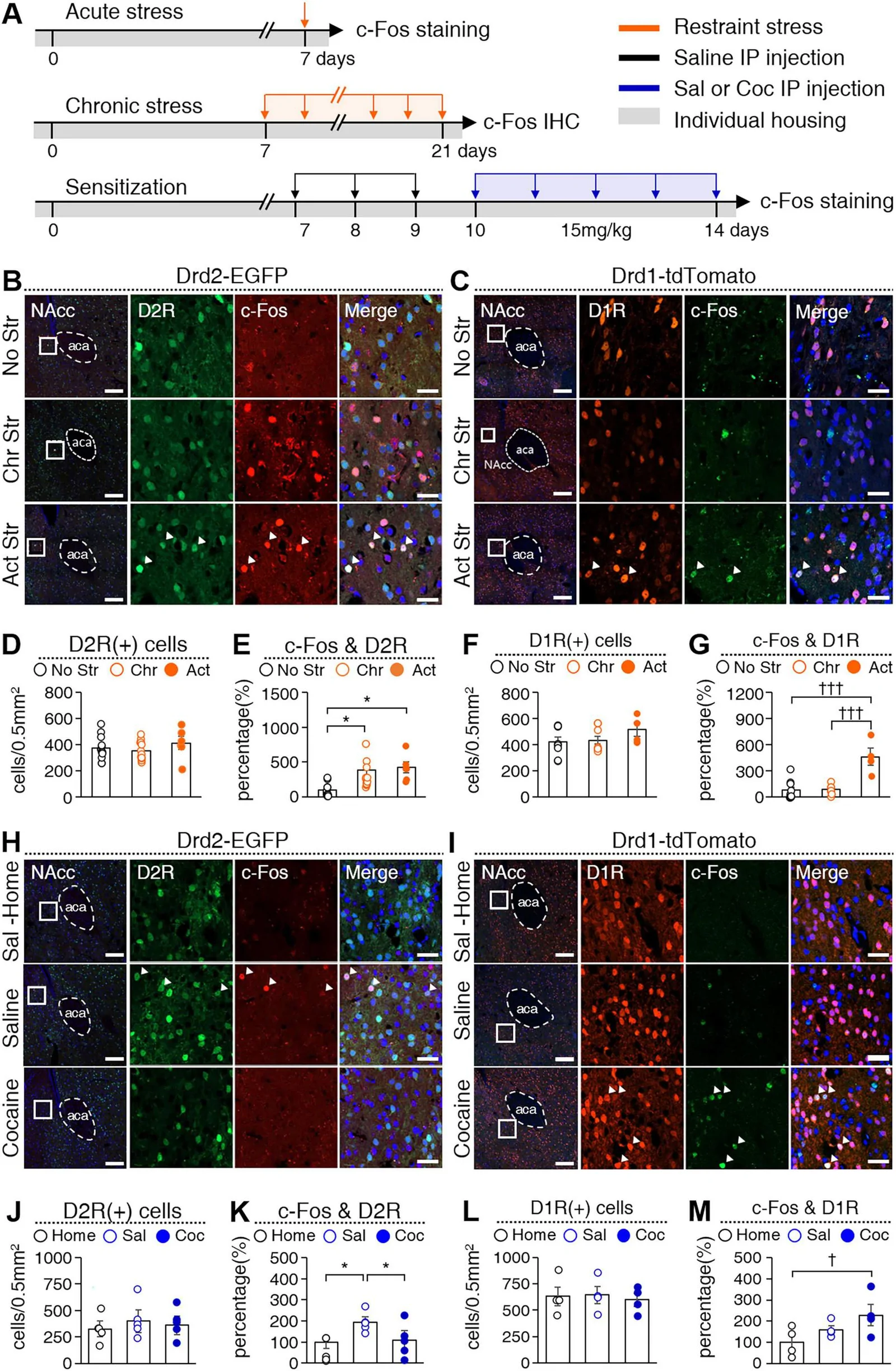
Neuronal activity of NAcc D2R-MSNs following acute restraint stress and repeated cocaine administration. **(A)** Experimental scheme for stress protocols and cocaine sensitization. **(B)** Representative images of c-Fos expression in the NAcc brain regions of *Drd2*-eGFP mice after restraint stress. Red: c-Fos, green: eGFP, blue: DAPI. Arrowheads indicate c-Fos & eGFP co-expressing cells, aca, anterior commissure (anterior part). Scale bar: low magnification, 100 μm, high magnification, 20 μm. **(C)** Representative images of c-Fos expression in the NAcc brain regions of *Drd1*-tdTomato mice following stress protocol. Arrowheads indicate c-Fos & tdTomato co-expressing cells, aca, anterior commissure (anterior part). Scale bar: low magnification, 100 μm, high magnification, 20 μm. **(D)** Quantification of eGFP-expressing D2R-MSNs in the NAcc (No stress group: *n* = 15, chronic stress group: *n* = 15, acute stress group: *n* = 6). **(E)** Percentage of c-Fos-positive cells within the D2R-MSN population. **(D, E)** Data are presented as mean ± standard error of the mean. One-way ANOVA, Tukey’s *post hoc*-test, *p < 0.05. All values in the data represent mean ± SEM. **(F)** Quantitative analysis of tdTomato-expressing D1R-MSNs in the NAcc (No stress group: *n* = 8, chronic stress group: *n* = 8, acute stress group: *n* = 5). **(G)** Percentage of c-Fos-expressing cells within the total D1R-MSN population. **(F, G)** Data are presented as mean ± standard error of the mean. One-way ANOVA, Tukey’s *post hoc*-test, ^†††^No Str vs. Act, *p <* 0.001. All values in the data represent mean ± SEM. **(H)** Representative images of c-Fos expression in the NAcc of *Drd2*-eGFP mice after initiation of cocaine sensitization. Red: c-Fos, green: eGFP, blue: DAPI. Arrowheads indicate c-Fos & eGFP co-expressing cells. Scale bar: low magnification, 100 μm, high magnification, 20 μm. **(I)** c-Fos expression in the NAcc brain regions of *Drd1*-tdTomato mice after initiation of cocaine sensitization protocol. Arrowheads indicate c-Fos & tdTomato co-expressing cells. Scale bar: low magnification, 100 μm, high magnification, 20 μm. **(J)** Quantification of eGFP-expressing D2R-MSNs in the NAcc (*n* = 5). **(K)** Percentage of c-Fos-positive cells within the D2R-MSN population after the cocaine exposure (*n* = 5). **(J, K)** Data are presented as mean ± standard error of the mean. One-way ANOVA, Tukey’s *post hoc*-test, *p < 0.05. All values in the data represent mean ± SEM. **(L)** Quantitative analysis of tdTomato-expressing D1R-MSNs in the NAcc (*n* = 4). **(M)** Percentage of c-Fos-expressing cells within the total D1R-MSN population (*n* = 4). **(L, M)** Data are presented as mean ± standard error of the mean. One-way ANOVA, Tukey’s *post hoc*-test, ^†^Home vs. Coc, *p <* 0.05. All values in the data represent mean ± SEM.

Mice received daily intraperitoneal (i.p.) injections of either saline or cocaine hydrochloride (15 mg/kg, Johnson Matthey Macfarlan Smith, UK) for 5 consecutive days. Following the 5-day initiation phase, mice underwent a 7-day withdrawal period in their home cages without any injections. For c-Fos immunohistochemistry experiments (Figure 1), mice underwent a 3-day saline pre-treatment phase (one daily i.p. saline injection) prior to the 5-day administration phase. Animals were then randomly assigned to receive either saline (Saline 3 days + Saline 5 days) or cocaine (Saline 3 days + Cocaine 5 days, 15 mg/kg/day i.p.) for 5 consecutive days. After the withdrawal period, to evaluate the expression of behavioral sensitization, mice were given a challenge injection of cocaine hydrochloride at a lower dose (10 mg/kg, i.p.) or saline, and their horizontal locomotor activity was recorded for an additional 30 min in the open-field chamber. Cocaine was dissolved in 0.9% NaCl. Immediately after each injection, horizontal locomotor activity was recorded for 30 min. This behavioral assessment was conducted using an open-field chamber (Med Associates, St. Albans, VT, USA). Behavioral testing and data acquisition were automatically recorded using the Activity Monitor software under predefined and standardized parameters to minimize experimenter bias.

### Cell type specific stress-activated D2R-MSNs marking system

AAV-RAM-d2TTA::TRE-FLEX-dTomato-WPREpA was injected bilaterally into the NAcc of Drd2-Cre mice. Mice were maintained on a doxycycline diet (Dox+; 40 ppm) to suppress reporter expression. Labeling was induced by subjecting mice to a 2-h acute restraint stress session under Dox-free (Dox−) conditions. Mice remained on the Dox- diet for 24 h post-stress, followed by a return to Dox+ chow. Subsequently, mice underwent a 5-day cocaine sensitization protocol (15 mg/kg, i.p.) under Dox+ conditions and were sacrificed 1 h after the final injection for c-Fos immunohistochemistry.

### *In vivo* optogenetic activation of stress-activated D2R-MSNs

Drd2-Cre mice were injected with pAAV-RAM-d2TTA::TRE-FLEX-ChR2-eYFP-WPREpA and implanted with optical fibers in the NAcc as described above. ChR2 expression was induced by acute restraint stress under Dox-free (Dox−) conditions; all subsequent procedures occurred under Dox+ conditions. Optical stimulation (473 nm) was delivered via a blue laser diode (Ultralasers, Quebec, Canada) controlled by a pulse generator (BNC 575). The stimulation protocol consisted of 20-Hz pulses (5-ms duration) at 2 mW for 1 h in their home cages. The laser power output was verified using a PM100D power meter (Thorlabs, Newton, NJ, USA).

### Immunofluorescence and confocal laser microscopy

Mice were anesthetized and perfused transcardially with PBS followed by 4% paraformaldehyde (PFA). Brains were post-fixed for 4 h, cryoprotected in 30% sucrose, and coronally sectioned at 40 μm using a cryostat Leica CM 1900 (Leica Microsystems, Wetzlar, Germany). Sections were pretreated with 1% NaOH/1% H*2*O*2* for 20 min and 0.3% glycine for 10 min, followed by blocking in 3% BSA and 0.3% Triton X-100. Sections were then incubated overnight at 4 °C with primary antibodies. The following primary antibodies were used: rabbit anti-Cre (1:200; LSBio, Seattle, WA, USA; LS-C179954), rabbit anti-c-Fos (1:100; Abcam, Cambridge, UK; ab190289), and rabbit anti-CCK (1:200; ImmunoStar, Hudson, WI, USA; #20078). After washing, sections were incubated with Alexa Fluor 568 or 488 goat anti-rabbit IgG secondary antibodies (1:500–1:1000) and counterstained with DAPI. Images were acquired using LSM 700 or LSM 800 confocal microscopes (Zeiss, Oberkochen, Germany) with a 40 × objective. Imaging parameters were kept constant across experimental groups.

### Quantitative analysis of c-Fos/CCK-immunoreactive cells

Cells were quantified in the NAcc (bregma +1.10 to +0.62 mm) across five sections per mouse. Counting frames (200 × 200 μm; 8 frames per hemisphere) were positioned around the anterior commissure. Only cells exhibiting clear somal fluorescence (c-Fos, CCK, GFP, or tdTomato) were included in the analysis. Image acquisition and subsequent quantitative cell counting were performed utilizing MetaMorph software (Molecular Devices, San Jose, CA, USA). To ensure objective and consistent quantification, automated thresholding based on fluorescence intensity and somal size filtering parameters was uniformly applied across all sections to identify and enumerate immunoreactive puncta.

### NAcc tissue dissection and dissociation

To achieve precise anatomical segregation between the nucleus accumbens core and shell while preserving optimal cell viability and RNA integrity, a specialized microdissection and enzymatic dissociation pipeline was employed. Mice were deeply anesthetized and transcardially perfused with ice-chilled NMDG-ACSF (93 mM NMDG, 2.5 mM KCl, 1.2 mM NaH_2_PO_4_⋅H_2_O, 30 mM NaHCO_3_, 20 mM HEPES, and 25 mM Glucose, pH 7.3–7.4) supplemented with 1 mM kynurenic acid, sodium L-ascorbate, thiourea, sodium pyruvate, 10 mM MgSO_4_, and 0.5 mM CaCl_2_ within 3 min to minimize ischemic and mechanical stress. Extracted brains were immediately sectioned into 0.5-mm-thick coronal slices utilizing a pre-chilled stainless steel coronal brain matrix (RWD Life Science, Shenzhen, China; 0.5-mm spacing) and razor blades. Bilateral NAcc tissue regions (Bregma +1.70 mm to +0.70 mm) were meticulously isolated using a 1-mm diameter sample punch (Harris Micro-Punch, Whatman, UK) under a stereomicroscope, avoiding adjacent nucleus accumbens shell or anterior commissure boundaries. RNA samples obtained from 6 mice per group were pooled by combining 3 mice per sample, yielding 2 biological replicates per group for downstream bulk RNA sequencing. NAcc tissues were dissociated using 80 U/mL papain (Worthington Biochemical, Lakewood, NJ, USA; LS003126) and 2.5 % (w/v) trehalose under continuous oxygenation via gentle 5 % CO_2_ bubbling. Mechanical trituration was performed sequentially using descending pipette tip diameters (2 mm, 1 mm, and uncut 1 mL tips; 50 strokes each) to fully dissociate the tissue into a single-cell suspension, which was then filtered through a 40 μm cell strainer. Following myelin clearance via magnetic beads (Miltenyi Biotec, Bergisch Gladbach, Germany; 130-096-733), the single-cell suspension was subjected to fluorescence-activated cell sorting (FACS) utilizing a FACSAria II flow cytometer (BD Biosciences, San Jose, CA, USA) with stringent gating parameters. To establish stringent gating parameters, a brain sample from a non-injected control mouse was utilized to define the threshold for background autofluorescence. Cells were sequentially gated based on forward scatter (FSC) and side scatter (SSC) profiles to exclude debris and doublets, followed by the isolation of the viable cell population which exhibited a viability of approximately 90 %. ChR2-eYFP-positive stress-activated D2R-MSNs were selectively isolated into RNAlater (Invitrogen, Carlsbad, CA, USA; AM7021)

### RNA sequencing and candidate gene selection

Total RNA was extracted from the FACS-isolated cells using the RNeasy Mini Kit (Qiagen, Hilden, Germany; 74104). To ensure sufficient RNA yield from sparse activity-tagged neurons, equal amounts of RNA extracted from 3 mice were pooled to generate 1 sample, yielding 2 biological replicates per group for bulk RNA sequencing (*n* = 2 per group, pooled from 3 mice each). RNA libraries were prepared using the SMARTer Ultra-low input kit and sequenced on an Illumina NovaSeq 6000 (101 bp paired-end). Reads were aligned to the mm10 reference genome using HISAT2, and gene expression was quantified via StringTie. The raw and individual normalized sequencing datasets for all biological replicates (*n* = 2 per group, total 4 samples) generated in this study have been deposited in the NCBI Gene Expression Omnibus (GEO) database under accession number GSE334247. The processed dataset of all detected genes, including group-mean normalized expression values and their corresponding fold changes, raw p-values, and FDR-adjusted p-values, is provided in Supplementary Table 1. Differential expression analysis was performed using edgeR. To isolate key differentially expressed genes from Supplementary Table 1, stricter criteria of fold change | log_2_FC| > 1 and FDR* <* 0.05 were applied. Because the statistical power for false discovery rate (FDR) estimation is inherently restricted by *n* = 2 biological replicates, this sequencing analysis was interpreted strictly as a preliminary screening criterion, consistent with previous studies employing limited biological replicates or pooled samples for exploratory omics profiling (Loeser et al., 2013; Reed et al., 2022). To rigorously refine primary candidates from this initial pool and mitigate potential false positives, we subsequently established a *post-hoc* bioinformatic filtering pipeline with clinical and translational relevance, adapting the computational framework described by Vannan et al. (2023). First, human homologs of the identified mouse DEGs were determined using the Ensembl database. Second, evolutionary conservation was assessed based on Gene Order Conservation (GOC) and Whole Genome Alignment (WGA) scores to filter for targets with high genomic fidelity between rodents and humans. Third, the selected candidates were cross-referenced with the Genotype-Tissue Expression (GTEx) portal database to evaluate their baseline expression profiles across human brain tissues, specifically restricting the final selection to genes dynamically expressed within the human nucleus accumbens. Validation was performed using qPCR and immunohistochemistry.

### Quantitative real-time PCR (qPCR)

Tissue dissection, fluorescence-activated cell sorting (FACS) of ChR2-eYFP-positive D2R-MSNs, and total RNA extraction were performed as described in the previous sections (“NAcc tissue dissection and dissociation” and “RNA Sequencing and Candidate Gene Selection”). For cDNA synthesis, equal amounts of the extracted total RNA were reverse-transcribed using Maxima Reverse Transcriptase (Fermentas, Waltham, MA, USA; EP0742) according to the manufacturer’s protocol. Real-time PCR was subsequently performed using LightCycler 480 SYBR Green I Master (Roche, Basel, Switzerland; 04887352001) on a CFX384 Real-Time PCR Detection System (Bio-Rad, Hercules, CA, USA). The PCR amplification was initiated with a denaturation at 95 °C for 10 min, followed by a touchdown cycling protocol where the annealing temperature decreased by 0.5 °C per cycle, before continuing with 45 standard cycles of 95 °C for 15 s and 60 °C for 1 min, including a final melting curve analysis. The relative expression levels of target genes were normalized to Gapdh as an internal loading control and calculated using the comparative 2^−ΔΔ*C*_*T*_^ method. The specific primer sequences used for qPCR were as follows (5’ to 3’):

- Ptger1, F: TGTGCGCTGTCTACGCTAAT, R: GCCTAGAC ACCACCAAGAAGC
- Wnt5b, F: CCTTCGCCCAGGTTGTTATA, R: GTTGCTGCA GCTTCGTTG
- Ntrk1, F: TTCCCGGAACGAAGGTAA, R: GTCCACGAA GAGCCAACAAC
- Nab2, F: CACCTACCCTGTCGGTGTTT, R: CTGGCGAGA GAAAGCTGGT
- Kcnj6, F: GGCTGGAGGACTTTGAGATG, R: ATCACCAC GATGATGAGGAA
- Cck, F: GAGCCGAAGCCTACAGGAT, R: CAAGAAGC GGGTTAGGTTGT
- Gapdh, F: AGGTCGGTGTGAACGGATTTG, R: TGT AGACCATGTAGTTGAGGTCA

### Statistical analysis

GraphPad Prism version 10 was used for statistical analyses. Data are presented as means ± SEM and were analyzed using the two-tailed Student’s *t-*test, with one-way or two-way ANOVA followed by either Tukey’s or Bonferroni’s *post-hoc* test. *p <* 0.05 was considered statistically significant.

## Results

### Restraint stress and repeated cocaine exposure differentially affect D2R-MSN activity

To test whether acute and chronic stress differentially modulate the activity of specific medium spiny neuron (MSN) subtypes within the NAcc, we examined c-Fos expression in *Drd1*-tdTomato and *Drd2*-EGFP transgenic mice following either a single 2-h acute restraint stress or chronic restraint stress (2 h daily for 2 weeks) (Figures 1A–C). Quantifications showed that the total numbers of D1R- and D2R-MSNs remained comparable across all experimental groups (Figures 1D,F). Both acute and chronic restraint stress increased c-Fos expression in D2R-MSNs (Figure 1E. *Drd2*-EGFP mice, no stress: 100.00 ± 22.73, chronic stress: 386.82 ± 99.57, acute stress: 423.65 ± 75.84). One-way ANOVA revealed a significant effect of the stress on c-Fos expression in *Drd2*-EGFP positive cells (*F*(2,33) = 5.44, *p* = 0.009). Tukey’s *post hoc*-test indicated that both acute restraint stress and chronic restraint stress resulted in significantly increased c-Fos expression compared to the no stress group (*p* < 0.05 for both). No significant difference was found between the acute stress and chronic stress groups (*p* > 0.05). In D1R-MSNs, acute restraint stress resulted in a significant increase in c-Fos expression compared to the no stress group, while chronic restraint stress did not induce any significant change (Figure 1G. *Drd1*-tdTomato mice, no stress: 100.00 ± 38.21, Chronic stress: 83.64 ± 19.20, acute stress: 459.73 ± 97.02). One-way ANOVA revealed a significant effect of the stress on c-Fos expression in D1R-tdTomato positive cells (*F*(2,17) = 17.14, *p* < 0.0001). Tukey’s *post hoc*-test showed that acute restraint stress resulted in a significant increase in c-Fos expression compared to both the no stress and chronic stress groups (*p* < 0.001 for both). In contrast, chronic restraint stress did not induce significant c-Fos activation in D1R-MSNs, as there was no significant difference observed between the no stress and chronic stress groups (*p* > 0.05).

Next, we examined NAcc neuronal activity following repeated cocaine administration (15 mg/kg, 5 days), which induced behavioral sensitization (Figures 1H,I). There was no significant difference in the total numbers of D1R- and D2R-MSNs among groups (Figures 1J,L). In D1R-MSNs, repeated cocaine elevated c-Fos expression compared to both home-cage and saline-chamber controls (Figure 1M. saline-home cage: 100.00 ± 7.55, saline-chamber: 159.14 ± 18.41, cocaine-chamber: 228.62 ± 50.00). One-way ANOVA revealed a significant effect of the cocaine administration and chamber exposure on c-Fos expression in NAcc D1R-MSNs (F(2, 9) = 4.29, *p* = 0.049). *Post hoc* analysis showed a significant difference between the home cage and cocaine groups (*p* < 0.05), indicating that D1R-MSNs were activated by repeated cocaine exposure.

In contrast, D2R-MSNs showed a distinct response pattern. Although saline injection and chamber exposure increased c-Fos expression relative to home-cage controls (*p <* 0.01), repeated cocaine administration significantly reduced c-Fos expression relative to the saline-treated group, returning activity to levels comparable to home-cage controls (Figure 1K. saline-home cage: 100.00 ± 28.86, saline-chamber: 193.65 ± 26.88, cocaine-chamber: 107.40 ± 45.62). One-way ANOVA revealed a significant effect of the cocaine administration and chamber exposure on c-Fos expression in NAcc D2R-MSNs (*F*(2, 11) = 10.09, *p* = 0.003). Tukey’s *post hoc*-test showed a significant difference between the Home and Saline groups, and also between the Saline and Cocaine groups, but not between home vs. cocaine. The effect of cocaine in this experiment is therefore best interpreted as a suppression of the elevated c-Fos activation observed following saline treatment, rather than a reduction below the home-cage baseline. These results suggested that NAcc MSN subtypes are differentially engaged by stress and drug experiences. D1R-MSNs were activated by cocaine, whereas D2R-MSNs were activated by stress and showed suppression of this activation following repeated cocaine exposure. To investigate how these stress signals modulate cocaine-induced addictive behaviors, we utilized the well-established acute restraint stress and subsequent cocaine behavioral sensitization paradigm (Kalivas and Stewart, 1991; Saal et al., 2003).

### *In vivo* fiber photometry reveals opposite modulation of D2R-MSNs by stress and cocaine

**FIGURE 2:**
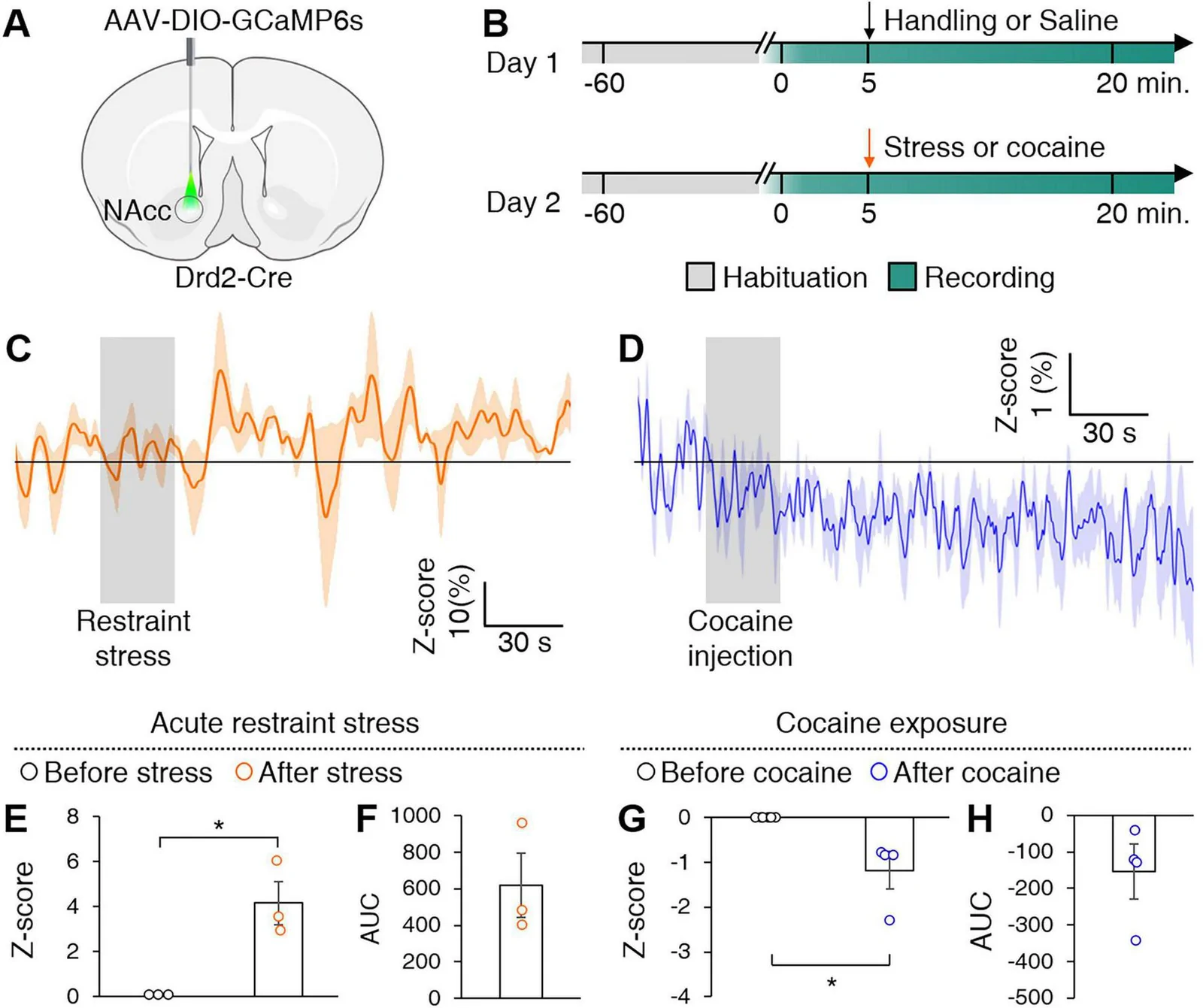
Modulation of NAcc D2R neuronal activity by restraint stress and cocaine exposure. **(A)** Diagram of AAV-DIO-GCaMP6s injection into the NAcc of *Drd2*-Cre mice. **(B)** Experimental timeline for *in vivo* fiber photometry recording during stress or cocaine exposure (15 mg/kg, IP). **(C)** Normalized z-score during restraint stress (*n* = 3). **(D)** Normalized z-score during cocaine exposure (IP injection 15 mg/kg, *n* = 4). **(E)** Average z-score pre- (-30 to 0 s) and post- (+30 to +180 s) restraint stress (*n* = 3). **(F)** AUC of post-restraint stress (+30 to +180 s). **(G)** Average z-score pre- (-30 to 0 s) and post- (+30 to +180 s) cocaine IP injection (*n* = 4). **(H)** AUC of post-cocaine IP injection (+30 to +180 s) (*n* = 4). **(E–H)** Data are presented as mean ± standard error of the mean. **(E, G)** Paired Student’s *t-*test, two-tailed, *p < 0.05.

To test whether the treatment-dependent c-Fos expression patterns reflect real-time neural dynamics in awake, behaving mice, we examined *in vivo* calcium transients in the NAcc D2R-MSNs during acute restraint stress and cocaine exposure using fiber photometry. A Cre-dependent calcium indicator (AAV-EF1a-DIO-GCaMP6s-P2A-nls-dTomato) was injected into the unilateral NAcc, followed by implantation of an optical fiber for *in vivo* recording (Figure 2A). We monitored real-time calcium transients before and after either restraint stress or cocaine exposure (Figure 2B). While fluorescence recordings were conducted on Day 1 to habituate the mice to the recording environment and handling/saline injection procedures, only the calcium signaling data obtained on Day 2 were used for the final quantitative analysis (Figures 2C,D). Histological analysis revealed that 97.06 ± 2.94% of GCaMP6-expressing cells overlapped with Cre-expressing NAcc D2R-MSNs (Supplementary Figures 1A–B). Calcium transients were recorded and analyzed as Z-scores to quantify the neuronal response to each treatment (Figures 2C,D). Consistent with our c-Fos results, D2R-MSN activity significantly increased following restraint stress (Figure 2E after Stress, mean Z-score: 4.16 ± 0.95, Student’s *t*-test, paired, two-tailed, *p = 0.0484; Figure 2F AUC: 621.02 ± 176.34), showing a prolonged increase in activity during acute restraint stress. In contrast, the calcium transients decreased after cocaine exposure (Figure 2G after Cocaine-injection, mean Z-score: −1.18 ± 0.42, Student’s *t*-test, paired, two-tailed, *p = 0.0490; Figure 2H. AUC: −153.78 ± 75.78). These results suggested that NAcc D2R-MSNs display bidirectional activity responses to environmental manipulations, characterized by sustained population activation under aversive stress and rapid neural suppression following psychostimulant exposure.

### Repeated cocaine exposure reduces the neuronal activity of stress-activated D2R-MSNs in the nucleus accumbens

**FIGURE 3:**
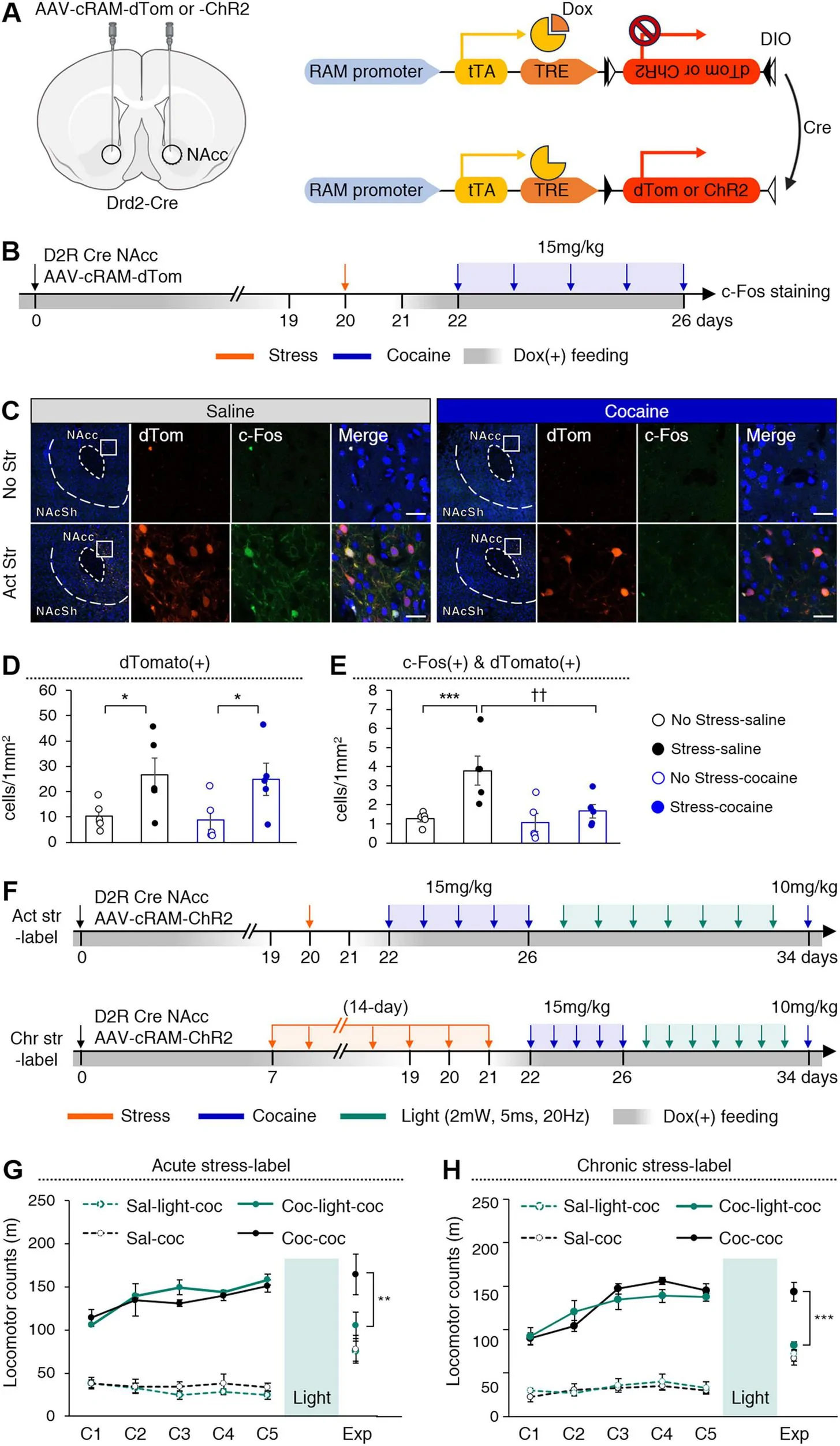
c-Fos expression in stress-activated D2R-MSNs within the NAcc. **(A)** Left: Diagram of AAV-cRAM-dTomato/ChR2-eYFP injection into the NAcc of *Drd2*-Cre mice (dTomato = dTom). Right: Diagram of the Cre- and Dox-dependent cRAM viral system. tTA, tetracycline-controlled transactivator; TRE, tetracycline response element; Dox, doxycycline; DIO, double-floxed inverse orientation; RAM, robust activity marking; AAV, adeno-associated virus; dTom, dTomato. **(B)** Experimental timeline for stress-activated D2R-MSN labeling and subsequent cocaine sensitization. **(C)** Representative images of c-Fos expression in stress-activated D2R-MSNs. Red: dTom, green: c-Fos, blue: DAPI. No Str: No stress, Act str: Acute restraint stress. NAcSh: Nucleus accumbens shell. Scale bar: 20 μm. **(D)** Quantification of dTomato-positive cells in the NAcc (*n* = 5, NoStr = No stress group, Str: Stress group, Sal: Saline group, Coc: Cocaine group). Student’s *t-*test, unpaired, two-tailed, *p < 0.05, NoStr vs. Str within each treatment group **(E)** The number of c-Fos and dTom double-positive cells relative to the total number of dTom-positive cells (*n* = 5). Two-way ANOVA, Bonferroni *post-hoc* test, ***NoStr-Sal vs. Str-Sal, *p <* 0.001, ^††^Str-Sal vs. Str-Coc, *p <* 0.01. **(F)** Experimental timeline for optogenetic stimulation during withdrawal following acute or chronic stress-labeling. (Act str-label: Acute stress-label scheme, Chr str-label: Chronic stress-label). **(G)** Locomotor activity during cocaine sensitization and expression following optogenetic activation of acute stress-activated D2R-MSNs during withdrawal. Sal−Coc: the group received saline for initiation, no light stimulation during withdrawal, and cocaine for expression. Sal-light−Coc: the group received saline for initiation, light stimulation during withdrawal, and cocaine for expression. Coc−Coc: the group received cocaine for initiation, no light stimulation during withdrawal, and cocaine for expression. Coc-light−Coc: the group received cocaine for initiation, light stimulation during withdrawal, and cocaine for expression (*n* = 4, C1–C5: Cocaine Day 1-5, Exp: Expression of sensitization). Two-way ANOVA, Bonferroni *post-hoc* test, **Coc-Coc vs. Coc-light−Coc, *p <* 0.01. **(H)** Locomotor activity following optogenetic activation of chronic stress-activated D2R-MSNs during the withdrawal period. Group designations are as described in **(G)** (*n* = 4). Two-way ANOVA, Bonferroni *post-hoc* test, ***Coc−Coc vs. Coc-light−Coc, *p <* 0.001.

To investigate whether the specific D2R-MSN subpopulation activated by restraint stress is subsequently inhibited by cocaine exposure, we utilized the cre-dependent Robust Activity Marking (cRAM) system (Sørensen et al., 2016). After labeling the acute restraint stress-activated D2R-MSN subpopulation with dTomato, mice were subjected to a repeated cocaine administration paradigm to induce behavioral sensitization (Figures 3A,B). First, to observe the labeling extent, we compared dTomato expression between stressed and non-stressed mice. Quantitative analysis revealed that restraint stress significantly increased the number of dTomato-positive D2R-MSNs compared with the no-stress group (Figures 3C,D. No stress-saline: 10.45 ± 2.46, Stress-saline: 26.55 ± 6.84, No stress-cocaine: 8.81 ± 3.81, Stress-cocaine: 24.87 ± 6.31; Student’s *t*-test, unpaired, two-tailed, *p* < 0.05). Two-way ANOVA on dTomato-positive cell density revealed a significant main effect of stress (F(1, 16) = 9.63, *p* = 0.007), but no main effect of cocaine treatment (F(1, 16) = 0.10, *p* = 0.753) or interaction (F(1, 16) < 0.01, *p* = 0.997). To further evaluate the effect of stress within saline or cocaine conditions, planned pair-wise comparisons were performed using an unpaired Student’s *t*-test.

We then investigated whether these specific stress-activated D2R-MSNs are modulated by subsequent cocaine exposure. We quantified cells co-expressing dTomato (indicating stress history) and c-Fos (indicating current activity state) following the final injection (Figure 3E). The number of dTomato and c-Fos double-positive cells decreased with repeated cocaine exposure. Two-way ANOVA revealed a significant main effect of stress (*F*(1,16) = 17.05, *p* < 0.001) and cocaine (*F*(1,16) = 10.11, *p* = 0.006) on the number of dTomato and c-Fos double-positive cells. The interaction between stress and cocaine was also significant (*F*(1,16) = 7.42, *p* = 0.015). *Post hoc* analysis showed that stress increased the number of dTomato and c-Fos double-positive cells in saline-treated mice (Figure 3E. No stress-saline: 1.26 ± 0.16, Stress-saline: 3.78 ± 0.76, *p <* 0.001). However, the number of dTomato and c-Fos double-positive cells was significantly attenuated by repeated cocaine administration (Figure 3E. Stress-saline: 3.78 ± 0.76, Stress-cocaine: 1.66 ± 0.36, *p <* 0.01). Furthermore, no difference was observed between stress and no-stress conditions in the cocaine group (Figure 3E. No stress-cocaine: 1.07 ± 0.46, Stress-cocaine: 1.66 ± 0.36, *p* > 0.05). Note that while cocaine suppresses the elevated activation of the total D2R-MSN population (Figure 1K), the lack of statistically significant suppression in non-stressed cRAM-tagged neurons (Figure 3E) reflects the limited number of tagged cells under the No Stress condition (Figure 3D), which reduces the sensitivity to detect further suppression. In summary, these findings suggested that repeated cocaine exposure is associated with suppressed activity within this specific population of stress-labeled D2R-MSNs.

### Optogenetic activation of acute stress-activated D2R-MSNs during withdrawal period reduces the expression of cocaine sensitization

To test whether repeated activation of stress-activated D2R-MSNs during withdrawal attenuates the expression of behavioral sensitization, we examined locomotor responses following selective optogenetic stimulation of this functional subpopulation. To test this, the AAV-cRAM-ChR2-eYFP viral vector was injected into the NAcc of *Drd2*-Cre mice, to drive Channelrhodopsin-2 (ChR2) expression specifically in neurons activated by stress (Figure 3A). Following a restraint stress protocol to induce ChR2 expression in stress-activated D2R-MSNs, we conducted a cocaine sensitization protocol (Figure 3F), during which mice received daily optogenetic stimulation during the withdrawal period (1 h/day; 20 Hz, 5 ms pulse width, 473 nm, 2 mW). Successful expression of stress-induced ChR2 in the NAcc was verified by fluorescence imaging (Supplementary Figure 2).

To systematically evaluate the role of optogenetic intervention during the withdrawal phase, mice were divided into four groups based on the combinations of drug treatments and light stimulation: Sal -Coc, Sal-Light-Coc, Coc-Coc, and Coc-Light-Coc (named in the order of initiation treatment - withdrawal phase stimulation – expression challenge). Optogenetic activation of stress-tagged D2R-MSNs during the withdrawal phase significantly reduced the expression of cocaine sensitization (Figure 3G. Coc*-*Coc: 164.52 ± 23.37, Coc-Light*-*Coc: 105.33 ± 15.57). Two-way ANOVA revealed a significant main effect of cocaine (*F*(1,12) = 22.34, *p* < 0.001) and light (*F*(1,12) = 8.86, *p* = 0.012) on the expression of sensitization. The interaction between cocaine and light was also significant (*F*(1,12) = 5.55, *p* = 0.036), indicating that the effect of optogenetic stimulation was specific to the cocaine-experienced group. Bonferroni *post-hoc* test showed that optogenetic activation significantly reduced the expression of sensitization compared to the no light condition, specifically in the cocaine group (*p <* 0.01). These results suggested that the activation of stress-responsive D2R-MSNs during withdrawal can attenuate the expression of cocaine sensitization.

### *In vivo* functional validation: chronic stress-activated D2R-MSNs modulate cocaine-induced sensitization

Given that drug addiction typically develops against a backdrop of prolonged, cumulative stress rather than a single isolated event, we next sought to extend our findings to a more clinically relevant pathology (Koob and Le Moal, 2001; Sinha, 2008). To test whether the NAcc D2R-MSN subpopulation activated by chronic stress similarly modulates drug-induced neuroadaptations, we examined behavioral responses following optogenetic activation of these chronic stress-activated D2R-MSNs during the withdrawal period (Figure 3F). Similar to the observations in the acute stress model, optogenetic activation during withdrawal specifically targeting the chronic stress-activated D2R-MSNs led to a reduction in expression of sensitization (Figure 3H, Sal-Coc: 67.30 ± 8.17, Sal-Light-Coc: 72.42 ± 8.56, Coc-Coc: 143.18 ± 10.85, Coc-Light-Coc: 81.56 ± 4.60). A two-way ANOVA revealed a significant main effect of cocaine (F(1,12) = 25.92, *p* < 0.001) and light (F(1,12) = 11.45, *p* = 0.005) on the expression of sensitization. The interaction between cocaine and light was also significant (F(1,12) = 15.97, *p* = 0.002). Bonferroni *post-hoc* test showed a significant increase in sensitization expression in the cocaine group compared to the saline group under the no light condition (*p* < 0.001), but no significant difference was observed under the light condition (*p* > 0.05). Additionally, Coc-Light-Coc group showed significant reduction of the expression of sensitization compared to Coc-Coc group (*p* < 0.001). No significant difference was observed between the light and no light conditions in the saline group, suggesting that activation of these neurons does not alter baseline locomotion but specifically counteracts cocaine-induced hyperlocomotion. These results suggested that D2R-MSNs activated by chronic stress also modulate cocaine-induced behavioral changes. However, it remains to be determined whether the neuronal subpopulations recruited by acute and chronic stress represent the same functional population.

### Transcriptomic analysis identifies CCK as a differentially regulated gene in acute stress-activated D2R-MSNs

**FIGURE 4:**
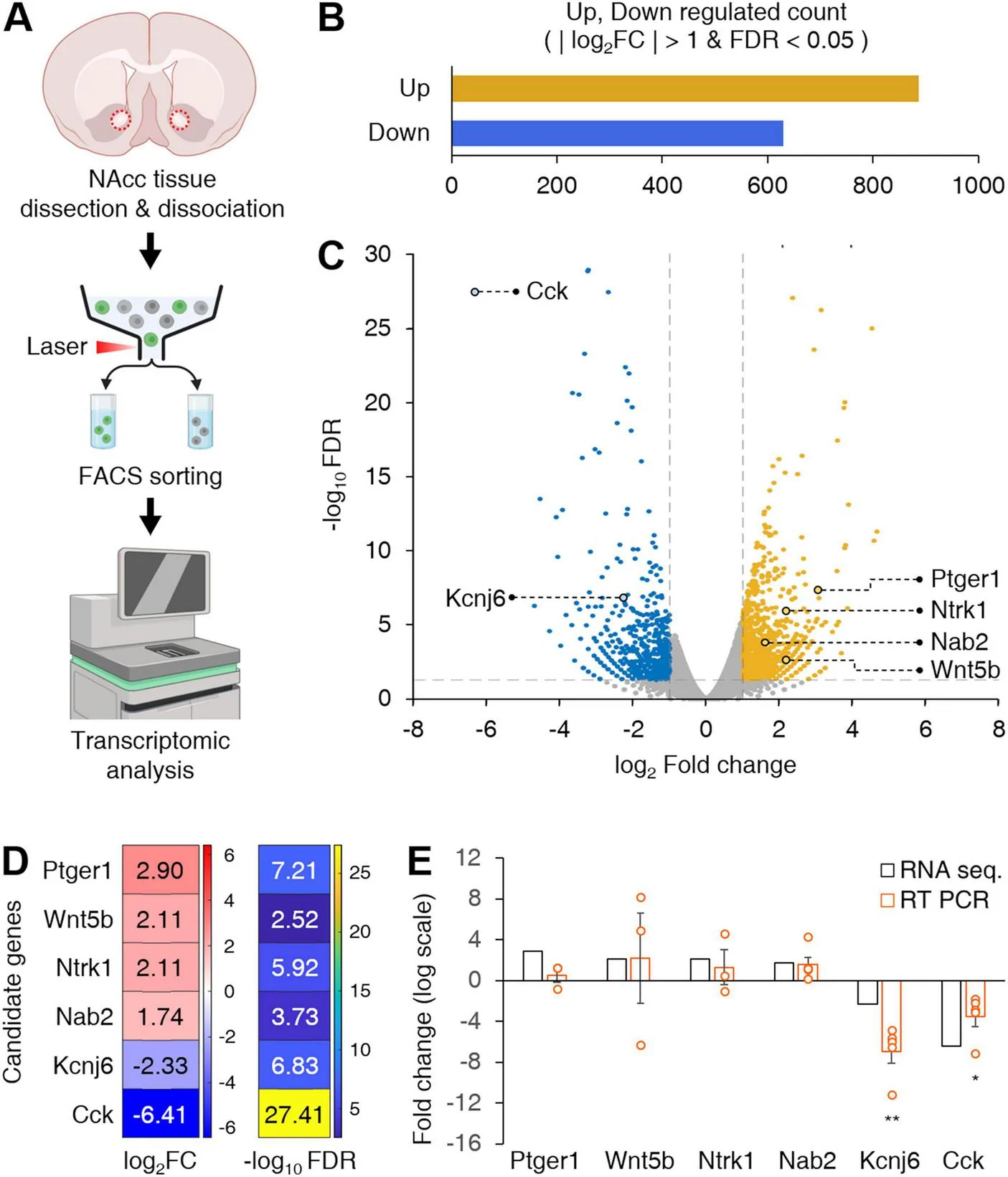
Transcriptomic profile of stress-activated NAcc D2R-MSNs following optogenetic stimulation during withdrawal. **(A)** Experimental workflow for FACS isolation and bulk RNA sequencing. **(B)** Total number of up- and down-regulated differentially expressed genes (DEGs) between Coc−Coc group and Coc-Light−Coc group (*n* = 2 biological replicates per group, pooled from 3 mice per sample). **(C)** Volcano plot of identified DEGs. (Yellow dots: upregulated genes, blue dots: downregulated genes, dashed vertical lines: | log_2_FC | >1, dashed horizontal line: statistical significance FDR* <* 0.05). **(D)** Heatmap of the fold change and statistical significance (-log_10_FDR) of selected candidate genes. (Left panel: fold change; red: upregulation, blue: downregulation. Right panel: statistical significance; color intensity: degree of significance). **(E)** Relative mRNA expression levels of selected candidate genes measured by real-time PCR (Student’s *t-*test, unpaired, two-tailed, *p < 0.05, **p < 0.01, *Ptger1*: prostaglandin E receptor 1, *Wnt5b*: Wnt family member 5B, *Ntrk1*: neurotrophic tyrosine kinase, receptor, type 1, *Nab2*: NGFI-A binding protein 2, *Kcnj6*: potassium inwardly rectifying channel subfamily J member 6, *Cck*: cholecystokinin, Sample sizes: *n* = 3 for *Ptger1*, *Wnt5b*, and *Ntrk1*, *n* = 5 for *Nab2*, *Kcnj6*, and *Cck*).

To test whether optogenetic reversal of cocaine sensitization induces specific molecular alterations within stress-activated NAcc D2R-MSNs, we examined potential transcriptional changes in these neurons. To identify differentially expressed genes associated with the reduction in cocaine sensitization, we conducted bulk RNA sequencing analysis on FACS-isolated neurons from the NAcc (Figure 4A). To assess the behavioral response in the cohort used for sequencing, we examined the effect of optogenetic manipulation on cocaine sensitization. Optogenetic activation of stress-activated D2R-MSNs during the withdrawal period significantly reduced the expression of sensitization, consistent with our previous observations (Supplementary Figure 3C. Expression of sensitization: Coc-Coc: 153.91 ± 10.54, Coc-Light-Coc: 92.63 ± 13.08). An unpaired *t*-test showed a significant difference between the two groups (t(10) = 3.65, *p* = 0.005). We then obtained ChR2-eYFP-positive stress-activated D2R-MSNs via FACS for RNA sequencing.

To evaluate the cell-type enrichment and consistency of the isolated samples, we examined the Transcripts Per Million (TPM) values of canonical MSN markers across groups. In the Coc-Coc (No Light) group, expression levels were high for D2R markers (*Drd2*: 160.72, *Penk*: 5,268.34 TPM) but low for D1R markers (*Drd1*: 47.07, *Pdyn*: 414.59 TPM). Similarly, the Coc-Light-Coc (Light) group showed high D2R marker expression (*Drd2*: 221.97, *Penk*: 6,293.20 TPM) with low D1R marker levels (*Drd1*: 59.54, *Pdyn*: 395.86 TPM). This consistent profile showed the predominant D2R-MSN identity and the technical reliability of the FACS isolation process. Notably, while the absolute expression of *Drd2* was lower than that of *Penk*, this difference aligned with the typical transcriptomic profiles of striatal MSNs, where GPCRs generally exhibit lower baseline transcription compared to highly abundant neuropeptide precursors (Gokce et al., 2016; Smith et al., 2019).

Differential expression analysis was conducted using a threshold of | log_2_FC| > 1 and a Benjamini–Hochberg adjusted *p*-value (FDR) < 0.05. Under these criteria, we identified a set of differentially expressed genes (DEGs) in the light*-*stimulated group compared with unstimulated control group (Figure 4B,C).

To systematically isolate the primary biological drivers from this unbiased dataset, we subsequently applied a data-driven, *post-hoc* filtering pipeline (see “Materials and methods”). Beyond statistical significance, candidates were filtered based on genomic conservation and NAcc-specific expression patterns to ensure biological relevance. As illustrated in the heatmap (Figure 4D), several key DEGs were identified through *post-hoc* screening process. Among the upregulated genes, *Ptger1* (log_2_FC = 2.90, −log_10_FDR = 7.21), *Wnt5b* (log_2_FC = 2.11, −log_10_FDR = 2.52), and *Ntrk1* (log_2_FC = 2.11, −log_10_FDR = 5.92) showed significant increases, alongside *Nab2* (log_2_FC = 1.74, −log_10_FDR = 3.73). Regarding downregulated genes, *Kcnj6* (log_2_FC = −2.33, −log_10_FDR = 6.83) and *Cck* (log_2_FC = −6.41, −log_10_FDR = 27.41) were identified. Among these, *Cck* exhibited the most robust fold change and the highest statistical significance.

The expression trends of selected genes were further examined by quantitative PCR (qPCR). qPCR analysis showed increased expression of *Ptger1*, *Wnt5b*, *Ntrk1*, and *Nab2*, and decreased expression of *Kcnj6* and *Cck*, consistent with the RNA-seq results (Figure 4E). In particular, significant decreases were observed in the transcript levels of *Kcnj6* and *Cck* (unpaired Student’s *t-*test, two-tailed; *p* = 0.0095 for *Kcnj6*, *p* = 0.0327 for *Cck*). Taken together, these observations suggested *Nab2, Kcnj6*, and *Cck* as the most promising candidates for further investigation.

### Cocaine exposure enhances CCK expression in NAcc D2R-MSNs

**FIGURE 5:**
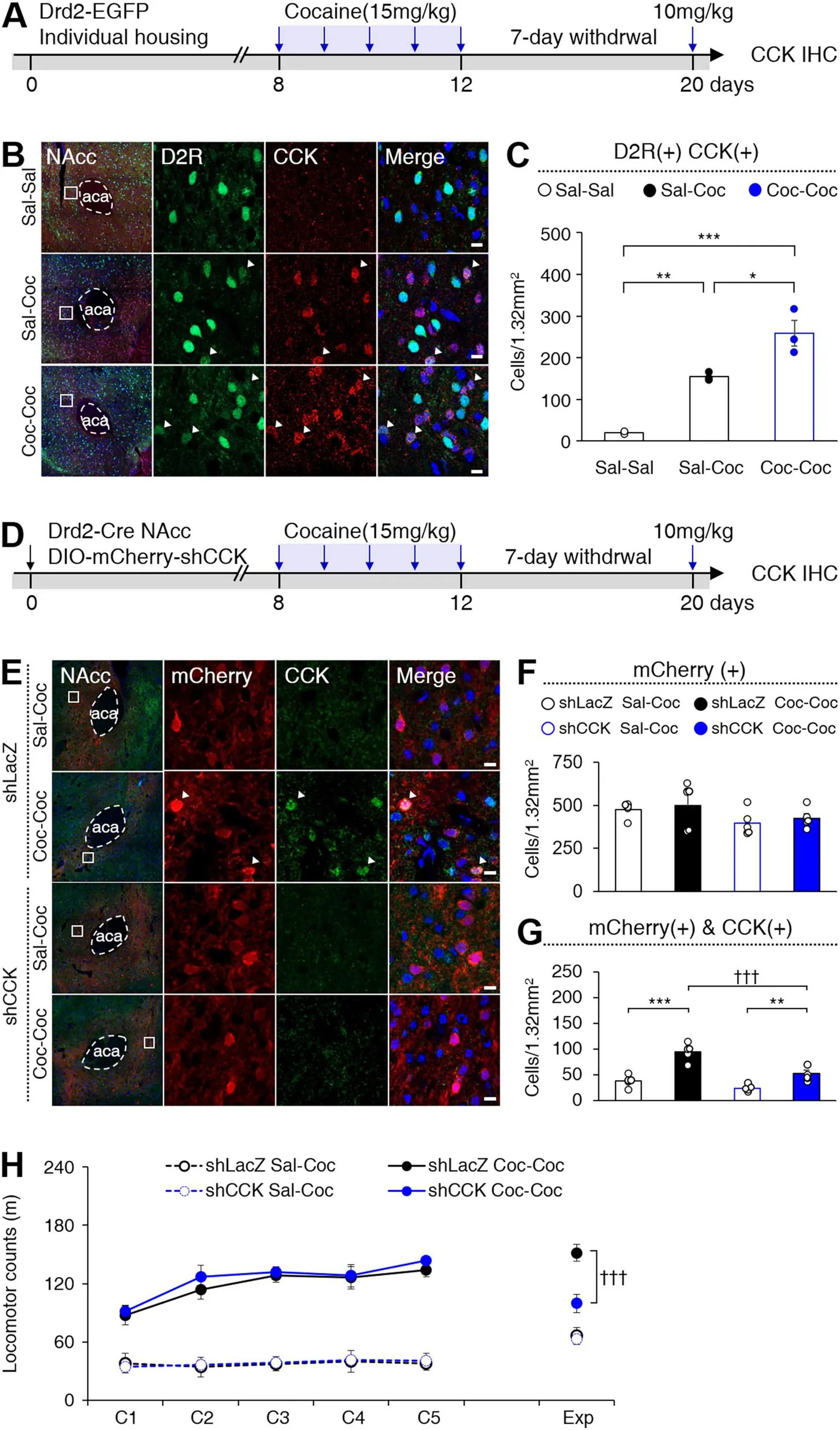
CCK expression in NAcc D2R-MSNs and behavioral effects of conditional CCK knockdown. **(A)** Experimental timeline for cocaine sensitization and subsequent CCK immunohistochemistry (IHC) in *Drd2*-EGFP mice. **(B)** Representative images of CCK expression within NAcc D2R-MSNs. Red: CCK, green: eGFP, blue: DAPI. Arrowheads indicate CCK & eGFP co-expressing cells. aca, anterior commissure (anterior part), Scale bar: 10 μm. **(C)** Quantification of CCK-positive D2R-MSNs following cocaine exposure (*n* = 3). One-way ANOVA, Tukey’s *post hoc*-test, *p < 0.05, **p < 0.01, ***p < 0.001. **(D)** Experimental scheme and timeline for conditional CCK knockdown and cocaine sensitization. **(E)** Representative images of CCK expression in the NAcc D2R-MSNs. Red: CCK, green: eGFP, blue: DAPI. Arrowheads indicate CCK & mCherry co-expressing cells. Scale bar: 10 μm. **(F-G)** Cell counts of D2R- and CCK-positive cells in the NAcc (*n* = 5). Sal-Coc: the group received saline for initiation and cocaine for expression. Coc-Coc: the group received cocaine for initiation and cocaine for expression. Two-way ANOVA, Bonferroni *post-hoc* test, **shCCK Sal-Coc vs. shCCK Coc-Coc, *p <* 0.01. ***shLacZ Sal-Coc vs. shLacZ Coc-Coc, *p <* 0.001. ^†††^shLacZ Coc-Coc vs. shCCK Coc- Coc, *p <* 0.001. **(H)** Locomotor activity during cocaine sensitization. (*n* = 5, C1–C5: Cocaine Day 1–5, Exp: Expression of sensitization). Two-way ANOVA, Bonferroni *post-hoc* test, ^†††^shLacZ Coc-Coc vs. shCCK Coc-Coc, *p <* 0.001.

To prioritize the final candidate gene for functional studies, we first investigated the protein-level expression and cell-type specificity of the selected candidates using immunohistochemistry on NAcc tissue (Figures 5A,B, Supplementary Figure 4). According to our prioritization pipeline based on genomic conservation, *Nab2* and *Kcnj6* (GIRK2) were initially prioritized as top-tier candidates due to their perfect evolutionary conservation scores (GOC = 100, WGA = 100 for both). In contrast, *Cck* possessed a slightly lower alignment profile (GOC = 100, WGA = 94.39). Although NAB2 and GIRK2 were detected in the D2R-MSNs, the majority of NAB2-expressing cells were non–D2R-MSNs, with only a small subset co-expressing D2R (Supplementary Figure 4B. 27.07 ± 0.31% of NAB2-expressing cells) and GIRK2 exhibited very low baseline expression in D2R-MSNs (Supplementary Figure 4C. 13.46 ± 5.00% of D2R-MSNs), suggesting low cell-type specificity or abundance. Given these limitations, we turned our attention to Cholecystokinin (*Cck*), which had recorded the most prominent transcriptomic alterations in our data. Public database profiling (GenSat^1^) and empirical observations indicated that *Cck* baseline expression is virtually scarce or negligible within the NAcc under drug-naive states. However, previous literature established that chronic cocaine exposure and subsequent behavioral sensitization trigger a progressive increase in extracellular CCK levels within the nucleus accumbens shell (Beinfeld et al., 2002) and distinctively induce striatal *Cck* mRNA transcription (Ding and Mocchetti, 1992). This literature-driven rationale suggested that while *Cck* remains dormant under basal conditions, it undergoes pathological induction specifically during cocaine-induced neuroadaptations. To test this drug-dependent induction at the cellular level, we systematically tracked the cell-type-specific protein regulation of CCK within NAcc D2R-MSNs across different stages of cocaine exposure using *Drd2*-EGFP mice (Figures 5A–C). Following the cocaine-induced behavioral sensitization (Figure 5A), we quantified CCK-immunoreactive cells. In the Sal-Coc group, a higher number of CCK-immunoreactive D2R-MSNs was observed compared to the Sal-Sal controls (Figures 5B,C. Sal-Sal: 20.67 ± 18.56, Sal-Coc: 154.67 ± 5.93). Furthermore, the Coc-Coc group, which underwent repeated cocaine exposure and 7-day withdrawal, showed a further increase in the number of CCK-positive D2R-MSNs (Figure 5C. Sal-Sal: 20.67 ± 18.56, Sal-Coc: 154.67 ± 5.93, Coc-Coc: 258.33 ± 30.59). One-way ANOVA showed a difference in the number of CCK-immunoreactive D2R-MSNs among the groups (*F*(2,6) = 43.73, *p* < 0.001). Tukey’s *post hoc*-test showed a higher number of these cells in the Sal-Coc group compared to the Sal-Sal group (*p <* 0.01). The number of CCK-positive D2R-MSNs was also higher in the Coc-Coc group compared to both the Sal-Sal group (*p <* 0.001) and the Sal-Coc group (*p <* 0.05). These results suggested that CCK protein expression within NAcc D2R-MSNs is upregulated by repeated cocaine exposure and subsequent drug withdrawal, establishing a rationale for further functional and causal analyses.

### Conditional knockdown of CCK in NAcc D2R-MSNs attenuates cocaine sensitization

To evaluate the functional necessity of CCK, we performed a conditional knockdown strategy targeting the D2R-MSN population within the NAcc. Due to the technical lack of an activity-dependent shRNA delivery system, this manipulation targeted the broader, virus-infected D2R-MSNs rather than the specific stress-activated subpopulation. We aimed to test whether broad CCK downregulation could recapitulate the behavioral suppression. To accomplish this, we delivered a shCCK viral vector (AAV-DIO-DSE-mCherry-PSE-shCCK) into the NAcc of *Drd2*-Cre mice (Figure 5D). Immunohistochemical analysis showed efficient knockdown, showing a significant reduction in cocaine-induced CCK expression within mCherry-positive neurons in the shCCK group compared with the shLacZ control (Figures 5E–G. shLacZ Sal-Coc: 38.25 ± 6.39, shLacZ Coc-Coc: 94.00 ± 9.86, shCCK Sal-Coc: 21.50 ± 1.71, shCCK Coc-Coc: 48.50 ± 5.52). Two-way ANOVA revealed a significant main effect of cocaine (*F*(1,16) = 55.76, *p <* 0.0001) and of shCCK (*F*(1,16) = 24.78, *p* < 0.001) on CCK expression. The interaction between cocaine and shCCK was also significant (*F*(1,16) = 6.02, *p* = 0.026). Bonferroni *post-hoc* test showed a significant decrease in cocaine-induced CCK expression in the shCCK group compared to the shLacZ control group (*p <* 0.01).

We then evaluated the behavioral responses following this conditional knockdown. In behavioral tests, CCK knockdown in D2R-MSNs did not affect cocaine-induced hyperlocomotion during the initiation of sensitization, which was comparable to the shLacZ control group (Figure 5H). However, following a 7-day withdrawal period, the shCCK group exhibited a lower expression of sensitization compared with controls (Figure 5H. shLacZ Sal-Coc: 66.28 ± 4.04, shLacZ Coc-Coc: 151.64 ± 8.73, shCCK Sal-Coc: 63.04 ± 4.16, shCCK Coc-Coc: 99.75 ± 9.38). Two-way ANOVA revealed a significant main effect of cocaine (*F*(1,16) = 75.35, *p <* 0.0001) and shCCK (*F*(1,16) = 15.37, *p* = 0.001) on the behavioral responses. The interaction between cocaine and shCCK was also significant (*F*(1,16) = 11.96, *p* = 0.003). Bonferroni *post-hoc* test showed that the shCCK group showed a significant reduction in cocaine-induced behavioral responses compared to the shLacZ control group under the cocaine condition (*p <* 0.001). No significant difference was observed between the shLacZ and shCCK groups under the saline condition (*p* > 0.05), suggesting that downregulation of CCK expression does not impair basal motor function. These results suggested that CCK signaling within NAcc D2R-MSNs is associated with the expression-but not the development*-*of cocaine behavioral sensitization following withdrawal.

## Discussion

In this study, we observed that NAcc D2R-MSNs exhibit distinct response patterns – activation by stress and suppression by repeated cocaine exposure. These findings align with previous reports demonstrating treatment-dependent modulation of D2R-MSN activity (Vialou et al., 2010; Francis et al., 2015; Calipari et al., 2016). Our findings also extend our previous work showing that D2R-mediated signaling plays an important role in adaptive responses to stress under drug-related conditions (Sim et al., 2013). Thus, D2R-MSNs are differentially engaged by stress and cocaine, providing a rationale for focusing on this neuronal population.

This differential modulation prompted us to examine whether a specific subpopulation of D2R-MSNs responds to both treatment. Activity-dependent labeling revealed that this subpopulation exhibits suppressed activity patterns following repeated cocaine exposure, suggesting stress-activated D2R-MSNs are particularly sensitive to subsequent cocaine-induced activity changes.

Consequently, we tested whether restoring the activity of these neurons might counteract cocaine-induced behavioral adaptations. Optogenetic activation of stress-activated D2R-MSNs during withdrawal attenuated the expression of behavioral sensitization. While previous studies have shown that activation of the broader D2R-MSN population reduces sensitization (Song et al., 2014), the present findings suggest that a functionally defined subpopulation may be particularly relevant to the suppression of cocaine-induced behavioral sensitization.

We next examined whether D2R-MSN activation by stress also influences behaviors under chronic conditions, given that chronic stress promotes addiction and alters mesolimbic dopamine transmission (Koob and Le Moal, 2001; Sinha, 2008; Miczek et al., 2008; Tidey and Miczek, 1997; Gambarana et al., 1999; Mangiavacchi et al., 2001). Optogenetic activation of chronic stress–activated D2R-MSNs during withdrawal suppressed the expression of cocaine-induced behavioral sensitization, similar to the effect observed following activation of acute stress–activated D2R-MSNs. These parallel behavioral outcomes suggest that recruiting stress-responsive D2R-MSNs – regardless of the stress duration – exerts an inhibitory effect on expression of sensitization.

However, a critical distinction must be made regarding the cellular identity of these recruited populations. Although both acute and chronic stress conditions produced comparable behavioral outcomes, it remains unclear whether they engage identical or distinct neuronal populations. While some stress-responsive populations can maintain functional identity over time (Balouek et al., 2023), chronic stress may recruit an expanded or distinct subset of D2R-MSNs due to prolonged neuroadaptations. Further investigation is required to determine the overlap between acute and chronic stress-responsive D2R-MSNs.

To explore the molecular mechanisms within these neurons, we profiled stress-activated D2R-MSNs and identified cholecystokinin (CCK). Although we focused on *Cck* due to its fold change, other prominent DEGs from our unbiased dataset, such as *Ptger1* (prostaglandin signaling) and *Wnt5b* (synaptic remodeling), also represent intriguing molecular candidates. While their functional roles remain to be elucidated, these unbiased transcriptomic profiles provide a valuable resource for discovering novel therapeutic targets in stress-drug interactions. CCK is a widely distributed neuropeptide that exerts diverse physiological functions across both the peripheral and central nervous systems (Crawley and Corwin, 1994). Peripherally, CCK acts primarily as a satiety peptide released by gastrointestinal cells to regulate digestion and food intake (Gibbs et al., 1973; Moran and Kinzig, 2004). In the central nervous system, CCK is one of the most abundant neuropeptides, functioning as a key neuromodulator involved in anxiety, nociception, learning, and reward processing (Harro et al., 1993; Ma and Giardino, 2022). Within the mesolimbic circuitry, CCK is prominently co-localized with dopamine in midbrain projections targeting the nucleus accumbens, where it acts via CCK1 (CCK_*A*_) and CCK2 (CCK_*B*_) receptors to bidirectionally modulate dopaminergic transmission, local circuit excitability, and synaptic plasticity (Hökfelt et al., 1980; Fallon and Seroogy, 1985; You et al., 1996; Ma and Giardino, 2022). Previous studies have reported interactions between CCK signaling and D2 receptor function. For example, deletion of CCK1R reduces D2R expression (Marco et al., 2012), and D2R stimulation increases striatal CCK mRNA levels (Ding and Mocchetti, 1992). Consistent with these findings, we observed that repeated cocaine exposure increased CCK expression in D2R-MSNs. Optogenetic activation of stress-activated D2R-MSNs down-regulated CCK expression alongside the attenuation of behavioral sensitization. This suggests that elevated CCK expression may be associated with cocaine sensitization, whereas its reduction suppresses this phenotype.

To further examine the potential contribution of CCK to this behavioral regulation, CCK expression was selectively reduced in D2R-MSNs. Although this manipulation targeted the broader D2R-MSN population due to the lack of tools for activity-dependent gene silencing, CCK knockdown mimicked the behavioral effects of optogenetic activation. However, because the manipulation was not restricted to stress-activated neurons, these results cannot determine whether CCK within the stress-activated D2R-MSN population specifically mediates this behavioral effect. Instead, the findings suggest that CCK signaling within D2R-MSNs may contribute to the regulation of cocaine sensitization. In this context, a critical question is whether the observed attenuation of cocaine sensitization via shCCK knockdown is uniquely driven by stress-activated D2R-MSNs or reflects a broader, state-dependent consequence of CCK suppression across the general NAcc D2R-MSN population. Extensive literature has established that endogenous CCK signaling within the NAcc plays a conditional role in modulating dopamine-mediated behaviors and psychostimulant responses. Specifically, systemic or intra-nucleus accumbens blockade of CCK_*A*_ receptors does not alter baseline or acute drug-induced locomotor activity in drug-naïve control animals (DeSousa et al., 1999; Wunderlich et al., 2004). However, following chronic psychostimulant exposure or chronic restraint stress, the mesoaccumbens CCK system becomes sensitized, such that intra-nucleus accumbens CCK receptor antagonism robustly attenuates the expression of behavioral sensitization as well as novelty-induced locomotion (DeSousa et al., 1999; Wunderlich et al., 2000, 2004). Therefore, we cannot entirely exclude the possibility that a general, random knockdown of CCK across total D2R-MSNs – independent of prior stress activation – exerts a general dampening effect on the expression of cocaine sensitization by blunting this sensitized neuropeptidergic tone. Nevertheless, the observation that CCK knockdown selectively blunted the expression of sensitization without altering initiation of sensitization indirectly implies that this behavioral regulation may depend on cocaine-induced plasticity rather than a non-specific, widespread suppression of the neural population. Given that CCK has been previously implicated in inhibitory synaptic plasticity (iLTP) within various GABAergic circuits (Chung and Moore, 2007, 2009; Crosby et al., 2018; He et al., 2023; Rice, 2023), a recent study by Guillaume et al. (2025) provides critical insights into the electrophysiological impact of CCK signaling within the striatum. Specifically, they demonstrated that blocking CCK2 receptors (CCK2R) lowers the action potential threshold and reduces the rheobase in medium spiny neurons, directly leading to increased intrinsic membrane excitability, while also shifting corticostriatal synaptic plasticity from long-term potentiation (LTP) to long-term depression (LTD). Considering these findings, our observation that optogenetic activation during withdrawal downregulates *Cck* expression in NAcc D2R-MSNs – and that shCCK knockdown mimics the suppression of cocaine sensitization – suggests that reducing CCK levels may alter intrinsic MSN excitability and recalibrate corticostriatal synaptic strength. In this context, a reduction in CCK tone following our manipulations might induce compensatory shifts in membrane excitability or plasticity rules within D2R-MSNs, thereby dampening the expression of drug-induced behavioral sensitization. Nevertheless, whether the alteration in CCK expression directly drives such cellular and circuit-level electrophysiological adaptations in our specific accumbens paradigm requires future single-cell electrophysiological validation.

Regarding the subcellular localization and release mode of CCK in NAcc D2R-MSNs, neuropeptides are synthesized in the soma and dendrites, processed, and selectively stored within large dense-core vesicles (LDCVs) (Martinez Damonte et al., 2023; Salio et al., 2006; van den Pol, 2012). These LDCVs are axonally transported to presynaptic nerve terminals and distributed within somatodendritic compartments (Martinez Damonte et al., 2023; Salio et al., 2006; van den Pol, 2012). In striatal and mesolimbic circuits, presynaptic localization of CCK—which can be co-localized with classical neurotransmitters such as dopamine or GABA—allows for activity-dependent co-release upon repetitive firing or depolarization, modulating local microcircuits and output targets (Hökfelt et al., 1980; Martinez Damonte et al., 2023; Salio et al., 2006; van den Pol, 2012). Beyond presynaptic release, CCK is also capable of being released from somatodendritic sites (Crosby et al., 2015; Martinez Damonte et al., 2023). Such somatodendritic peptide release can trigger autocrine or paracrine signaling cascades, activating postsynaptic receptors or liberating retrograde messengers to modulate synaptic strength and neuronal excitability (Crosby et al., 2015; Martinez Damonte et al., 2023). While detailed immunoelectron microscopic validation is required to map CCK-containing LDCVs in nucleus accumbens D2R-MSNs specifically, this dual axonal and somatodendritic distribution supports a multifaceted role for CCK in tuning local microcircuit dynamic balance and mediating broader neuromodulatory actions (Crosby et al., 2015; Martinez Damonte et al., 2023; Nadim and Bucher, 2014; Salio et al., 2006; van den Pol, 2012).

While nucleus accumbens CCK is historically considered to be primarily extrinsic, originating from VTA, BLA, and PFC projections (Ma and Giardino, 2022), our transcriptomic analysis identified Cck mRNA within stress-activated D2R-MSNs. Although local CCK expression in NAcc MSNs is exceptionally low under basal conditions (Figures 5B,C), our findings indicate that endogenous *Cck* transcripts are present in D2R-MSNs under cocaine-exposed conditions. This possibility is supported by our FACS-sorted RNA-seq and qPCR analyses and by the effects of local shCCK knockdown, which targets intracellular *Cck* mRNA transcripts. Furthermore, this capacity for local synthesis is aligned with prior evidence that striatal *Cck* mRNA expression is tonically regulated and induced via D2 receptor pathways upon cocaine exposure (Ding and Mocchetti, 1992). Nonetheless, intracellular CCK signals may reflect contributions from both endogenous *Cck* expression within D2R-MSNs and ligand-induced internalization of extracellular CCK–CCKR complexes. Future studies utilizing cell-specific translation assays versus blockade of receptor endocytosis will further clarify the relative contribution of each pathway to stress-induced behavioral adaptations.

In conclusion, our study demonstrates that a stress-activated D2R-MSN population is involved in stress-associated cocaine-related behaviors. Furthermore, our findings suggest that CCK may modulate sensitization expression within the D2R-MSN subpopulation. Together, these results highlight a potential link between stress-responsive neural activity and drug-related behavioral changes within a specific neural population.

## Study limitations

Despite these findings, several limitations must be acknowledged. First, while the cRAM system enables activity-dependent labeling, its efficacy depends on a restricted temporal window of induction. Consequently, it may capture only a subset of neurons active during that specific timeframe, rather than the entire, heterogeneous stress-responsive population, thereby limiting population specificity. Furthermore, the exact percentage of the total D2R-MSN population successfully captured by the cRAM system could not be determined in this study, as the total D2R-MSN pool was not concurrently quantified within the same experimental subjects. Second, our fiber photometry recordings capture aggregate calcium signals from a population of neurons. This bulk recording lacks single-cell resolution and cannot isolate individual firing patterns. Moreover, although the sample size for these fiber photometry experiments was relatively small (*n* = 3–4 per group), the observed calcium transients showed a consistent trend across all tested animals. However, given the limited statistical power inherent to a smaller cohort, further validation with a larger sample size will be necessary to confirm the generalizability of these findings. Third, although our transcriptomic profiling was performed on the isolated stress-activated D2R-MSN subpopulation, it relied on bulk RNA sequencing. Consequently, this approach yields an averaged gene expression profile that masks individual single-cell heterogeneity and potential distinct cell states within this functional subpopulation. Fourth, a direct causal link between CCK within the stress-activated subpopulation and behavioral regulation remains incomplete. Because current genetic tools lack the capacity for activity-dependent gene silencing, our CCK knockdown was broad and non-specific, leaving it unclear whether the attenuation of sensitization expression was driven specifically by CCK reduction within stress-activated D2R-MSNs or through broader network-wide suppression.

Furthermore, while the cocaine sensitization model provides valuable insights into neuroadaptations, it fails to capture the voluntary, motivational, and compulsive aspects of drug addiction. Therefore, future studies utilizing cocaine self-administration or reinstatement models are warranted to determine whether CCK genuinely regulates these voluntary drug-seeking behaviors. In addition, incorporating single-cell transcriptomic approaches within these behavioral paradigms will help verify whether the molecular changes we observed are conserved during the transition to compulsive drug taking. Moreover, dissecting the contributions of CCK receptor subtypes (CCK1R and CCK2R) will be essential. Furthermore, because this study was conducted exclusively in male mice to minimize potential variability, future investigations including female subjects are necessary to determine potential sex-dependent differences (Park et al., 2019). Finally, exploration of other candidate genes (e.g., *Nab2*, *Kcnj6*, *Wnt5b*) may reveal additional layers of molecular regulation. Collectively, addressing these limitations will broaden our understanding of the molecular substrates involved in stress-induced changes in drug-related behaviors.

Lastly, a technical limitation of our optogenetic approach is the absence of an opsin-free (AAV-cRAM-dTomato) control group receiving light stimulation. To ensure strict technical consistency and apply completely identical fluorescence gating parameters during the FACS-assisted sorting for downstream bulk RNA sequencing, we deliberately utilized identical AAV-cRAM-ChR2-eYFP expressions across both the experimental (Light) and control (No Light) cohorts, thereby eliminating potential sorting and isolation biases. While this design optimized the transcriptomic reliability of our isolated samples, it precludes the absolute exclusion of non-specific effects potentially induced by the laser stimulation itself, such as localized thermal shifts. Future studies incorporating opsin-free controls with identical illumination paradigms will be valuable to definitively rule out any confounding photostimulation effects.

## Data Availability

The datasets presented in this study can be found in online repositories. The names of the repository/repositories and accession number(s) can be found in the article/Supplementary material.

## Footnotes

1
https://www.gensat.org
