# Supplementary material for: Stress-responsive nucleus accumbens dopamine D2 receptor-expressing neurons modulate cocaine-induced behavioral sensitization
Source: Front Cell Neurosci. 2026 Aug 12;20:1919290. doi: 10.3389/fncel.2026.1919290 (PMC13506371; doi:10.3389/fncel.2026.1919290)
Supplement: Supplementary file 1 [file Supplementary_file_1.docx]

**Supplementary Material**

**Stress-responsive Nucleus Accumbens Dopamine D2 Receptor-expressing Neurons Modulate Cocaine-induced Behavioral Sensitization**

Byeong Jun Kang^1^, Bokyeong Kim^1^, Minji Kim^1^, Yoonsoo Choi^1^, Eum-Ji Kim^2^, Ja Wook Koo^2, 3^ and Ja-Hyun Baik^1†^

^1^Molecular Neurobiology Laboratory, Department of Life Sciences, School of Life Sciences and Biotechnology, Korea University, Seoul 02841, Republic of Korea.

^2^Emotion, Cognition and Behavior Research Group, Korea Brain Research Institute, Daegu 41062, Republic of Korea

^3^Department of Brain Sciences, Daegu Gyeongbuk Institute of Science and Technology, Daegu 41988, Republic of Korea.

†Corresponding author: Dr. Ja-Hyun Baik

Molecular Neurobiology Laboratory, Department of Life Sciences, School of Life Sciences and Biotechnology, Korea University, Seoul 02841, Republic of Korea.

This file contains:

- Supplementary Figures 1 to 4

- Supplementary Figure Captions

- Supplementary Table Captions

**Supplemental Figure 1.** **Viral expression of AAV-DIO-GCaMP6s in the NAcc of *Drd2*-Cre mice**

(A) Representative images of AAV-DIO-GCaMP6s virus expression in the NAcc at low magnification and colocalization of Cre- and GCaMP6s-positive cells in the NAcc. Red: Cre, green: GCaMP6s, blue: DAPI. aca, anterior commissure (anterior part). Scale bar: 20 μm. (B) Cell counts of Cre- and GCaMP6s-positive cells in the NAcc of *Drd2*-Cre mice (*n =* 3).

 **Supplemental figure 2.** **ChR2 expression of acute or chronic stress-activated-D2R-MSNs using cRAM system.**

(A) Representative images of ChR2 expression in the acute or chronic stress-responsive D2R-MSNs. green; ChR2-eYFP, blue; DAPI. aca, anterior commissure (anterior part). Scale bar: 20 μm. Sal-Coc: the group received saline for initiation and cocaine for expression. Coc-Coc: the group received cocaine for initiation and expression.

**Supplemental figure 3.** **Cocaine-induced behavioral sensitization of mice used for bulk RNA sequencing**

(A) Left: Diagram of AAV-cRAM-ChR2-eYFP injection into the NAcc of *Drd2*-Cre mice. Right: Diagram of the Cre- and Dox-dependent AAV-cRAM-ChR2-eYFP viral system. (B) A schematic diagram of subsequent experimental timeline of the labeling of stress-activated-D2R-MSNs with ChR2 and cocaine sensitization and optogenetic stimulation during the withdrawal period. (C) Locomotor activity during cocaine sensitization and expression following optic activation of chronic stress-activated D2R-MSNs during withdrawal. Coc-Coc: the group received cocaine for initiation, no light stimulation during withdrawal, and cocaine for expression. Coc-light-Coc: the group received cocaine for initiation, light stimulation during withdrawal, and cocaine for expression. (*n =* 6, C1-C5: Cocaine Day 1-5, Exp: Expression of sensitization). Unpaired student’s t-test, two-tailed, **: Coc -Coc vs Coc-light-Coc, *p =* 0.005

**Supplemental figure** **4.** **NAB2 and GIRK2 expression in NAcc D2R-MSNs**

(A) Representative images of NAB2 and GIRK2 expression in the NAcc D2R-MSNs of *Drd2*-EGFP mice. Red: NAB2 or GIRK2, green; eGFP, blue; DAPI. aca, anterior commissure (anterior part), White arrowheads indicate D2R & NAB2+ cells, yellow arrowheads indicate GIRK+ cells. Scale bar: 10 μm. (B) Quantification of D2R- and NAB2-positive cells in the NAcc (*n =* 3). (C) Quantification of D2R- and GIRK2-positive cells in the NAcc (*n =* 3).

**Supplementary Table Captions**

**Supplementary Table 1. Transcriptomic profile and differentially expressed genes (DEGs) of stress-activated NAcc D2R-MSNs.**

Processed RNA-sequencing dataset for all detected genes comparing the Coc-Coc (No_light) and Coc-Light-Coc (light_1) groups. (Coc-Coc: the group received cocaine for initiation, no light stimulation during withdrawal, and cocaine for expression; Coc-Light-Coc: the group received cocaine for initiation, light stimulation during withdrawal, and cocaine for expression.) The table provides normalized expression levels (FPKM and TPM), average expression magnitudes (logCPM), group-mean log_2_ fold changes (fc), raw p-values (raw.pval), and Benjamini-Hochberg FDR-adjusted p-values (bh.pval) for comprehensive review. Abbreviations: FPKM, Fragments Per Kilobase of transcript per Million mapped reads; TPM, Transcripts Per Million; fc, log_2_ Fold Change; logCPM, log_2_ Counts Per Million; raw.pval, Unadjusted *p-*value; bh.pval, Benjamini-Hochberg Adjusted *p-*value.
